# Modulation by Ozone Therapy of Oxidative Stress in Chemotherapy-Induced Peripheral Neuropathy: The Background for a Randomized Clinical Trial

**DOI:** 10.3390/ijms22062802

**Published:** 2021-03-10

**Authors:** Bernardino Clavo, Gregorio Martínez-Sánchez, Francisco Rodríguez-Esparragón, Delvys Rodríguez-Abreu, Saray Galván, David Aguiar-Bujanda, Juan A. Díaz-Garrido, Silvia Cañas, Laura B. Torres-Mata, Himar Fabelo, Teresa Téllez, Norberto Santana-Rodríguez, Leandro Fernández-Pérez, Gustavo Marrero-Callico

**Affiliations:** 1 Research Unit, Hospital Universitario de Gran Canaria Dr. Negrín, 35019 Las Palmas de Gran Canaria, Spain; frodesp@gobiernodecanarias.org (F.R.-E.); ltm1002@gmail.com (L.B.T.-M.); 2 Chronic Pain Unit, Dr. Negrín University Hospital, 35019 Las Palmas de Gran Canaria, Spain; 3 Radiation Oncology Department, Hospital Universitario Dr. Negrín, 35019 Las Palmas de Gran Canaria, Spain; 4 Universitary Institute for Research in Biomedicine and Health (iUIBS), Molecular and Translational Pharmacology Group, University of Las Palmas de Gran Canaria, 35016 Las Palmas de Gran Canaria, Spain; norbesanrod@gmail.com (N.S.-R.); leandrofco.fernandez@ulpgc.es (L.F.-P.); 5 Spanish Group of Clinical Research in Radiation Oncology (GICOR), 28290 Madrid, Spain; 6 Research Network on Health Services in Chronic Diseases (REDISSEC), Instituto de Salud Carlos III, 28029 Madrid, Spain; teresatellez@yahoo.com; 7 Scientific Advisor, Freelance, 60126 Ancona, Italy; gregorcuba@yahoo.it; 8 Medical Oncology Department, Complejo Hospitalario Universitario Insular Materno-Infantil de Gran Canaria, 35016 Las Palmas de Gran Canaria, Spain; drodabr@gobiernodecanarias.org; 9 Medical Oncology Department, Hospital Universitario de Gran Canaria Dr. Negrín, 35019 Las Palmas de Gran Canaria, Spain; saraygalrui@gmail.com (S.G.); dagubuj@gobiernodecanarias.org (D.A.-B.); 10 Psychiatry Department, Hospital Universitario de Gran Canaria Dr. Negrín, 35019 Las Palmas de Gran Canaria, Spain; juanantdiaz@hotmail.com; 11 Psychiatry Department, Complejo Hospitalario Universitario Insular Materno-Infantil de Gran Canaria, 35016 Las Palmas de Gran Canaria, Spain; silvia.cj1990@gmail.com; 12 Institute for Applied Microelectronics (IUMA), University of Las Palmas de Gran Canaria (ULPGC), 35017 Las Palmas de Gran Canaria, Spain; hfabelo@iuma.ulpgc.es (H.F.); gustavo@iuma.ulpgc.es (G.M.-C.); 13 Departamento de Especialidades Quirúrgicas, Bioquímica e Inmunología, Facultad de Medicina, Universidad de Málaga, 29010 Málaga, Spain; 14 Thoracic Surgery, Department of Surgery, King Faisal Specialist Hospital and Research Center, Riyadh 11564, Saudi Arabia; 15 College of Medicine, Department of Surgery, Alfaisal University, Riyadh 11533, Saudi Arabia

**Keywords:** antioxidants, cancer treatment, chemotherapy-induced peripheral neuropathy, chemotherapy-induced side effects, chemotherapy-induced toxicity, oxaliplatin, free radicals, oxidative stress, ozone therapy, randomized clinical trial

## Abstract

(1) Background: Chemotherapy-induced peripheral neuropathy (CIPN) decreases the quality of life of patients and can lead to a dose reduction and/or the interruption of chemotherapy treatment, limiting its effectiveness. Potential pathophysiological mechanisms involved in the pathogenesis of CIPN include chronic oxidative stress and subsequent increase in free radicals and proinflammatory cytokines. Approaches for the treatment of CIPN are highly limited in their number and efficacy, although several antioxidant-based therapies have been tried. On the other hand, ozone therapy can induce an adaptive antioxidant and anti-inflammatory response, which could be potentially useful in the management of CIPN. (2) Methods: The aims of this works are: (a) to summarize the potential mechanisms that could induce CIPN by the most relevant drugs (platinum, taxanes, vinca alkaloids, and bortezomib), with particular focus on the role of oxidative stress; (b) to summarize the current situation of prophylactic and treatment approaches; (c) to describe the action mechanisms of ozone therapy to modify oxidative stress and inflammation with its potential repercussions for CIPN; (d) to describe related experimental and clinical reports with ozone therapy in chemo-induced neurologic symptoms and CIPN; and (e) to show the main details about an ongoing focused clinical trial. (3) Results: A wide background relating to the mechanisms of action and a small number of experimental and clinical reports suggest that ozone therapy could be useful to prevent or improve CIPN. (4) Conclusions: Currently, there are no clinically relevant approaches for the prevention and treatment of stablished CIPN. The potential role of ozone therapy in this syndrome merits further research. Randomized controlled trials are ongoing.

## 1. Introduction

Due to advances in cancer treatment, the number of cancer survivors is increasing, and they are living longer and/or with better quality of life. However, as a consequence, the number of survivors with acute and/or chronic side effects of cancer treatments is also increasing. Development and assessment strategies to mitigate and manage chronic toxicities associated with cancer treatment have been established as an urgent area of research for the American Society of Clinical Oncology (ASCO) [1].

One of the most relevant cancer treatment toxicities is chemotherapy-induced peripheral neuropathy (CIPN), which can produce local alterations (dysesthesias and/or pain) and associated worsening of general symptoms (i.e., anxiety, depression, insomnia, fatigue), with high impact on patients’ quality of life. During cancer treatment, the relevance of these symptoms can reduce or delay the scheduled doses of chemotherapy, or even lead to its interruption, with the subsequent decrease in anti-tumoral efficacy. Between 19% and 85% of cancer patients treated with neurotoxic chemotherapy can develop CIPN, and 70–100% in platinum-based drugs [2]. This percentage could be increased by antiangiogenic drugs added to the new chemotherapy protocols [3]. Between 20% and 40% of these cancer patients could suffer pain secondary to CIPN [4]. Unfortunately, the approaches used for the management of other neuropathic pain syndromes do not work properly in CIPN [5] and there are no clinically relevant prophylactic or treatment approaches for CIPN [6,7,8].

The induction of reactive oxygen species (ROS) by chemotherapy drugs, with further oxidative stress (OS), is one of the potential pathogenic mechanisms that can induce CIPN [9], and this work is focused on this mechanism. However, there are other potential mechanisms of the induction of CIPN, many of which are highly related to OS, such as: damage in microtubules, myelin or DNA; local processes of inflammation, ischemia, or ion channel alterations in Na, K, or Ca; and transient receptor potential (TRP) channels [2,10], which show sensitivity to different endogenous molecules, including ROS [11].

In addition to many different approaches, anti-inflammatory drugs and several antioxidant therapies have been evaluated in the management of CIPN, although without conclusive results to date. Therefore, prophylactic and therapeutic options continue to be highly limited in number and efficacy [6,7,8].

The appropriate systemic administration of ozone has been well described as a potential inducer of an adaptive antioxidant response, with the further modulation of OS and inflammation related to several conditions and drugs, including chemotherapy drugs [12].

The aims of this works are: (a) to summarize the potential mechanisms that could induce CIPN by the most relevant drugs (platinum, taxanes, vinca alkaloids, and bortezomib), with particular focus on the role of oxidative stress; (b) to summarize the current situation of prophylactic and treatment approaches; (c) to describe the action mechanisms of ozone therapy to modify OS and inflammation with its potential repercussions for CIPN; (d) to describe experimental and clinical reports related to ozone therapy in chemo-induced neurologic symptoms and CIPN; (e) to show the main details about an ongoing focused clinical trial.

## 2. Chemotherapy-Induced Peripheral Neuropathy and Oxidative Stress

The mechanisms involved in the pathogenesis of CIPN are multifactorial and involve microtubule disruption, OS, and mitochondrial damage and apoptosis induced by ROS, myelin sheath damage, DNA damage, immunological processes, neuroinflammation and increased production of pro-inflammatory mediators linked to OS or to the action of chemotherapy drugs, and altered ion channel activity [2,13].

There are different mechanisms of ROS generation. One of the most important endogenous, unavoidable mechanisms is the production of ROS in mitochondria by oxidative phosphorylation. The pharmacological mechanisms of chemotherapy, at least in part, can be explained by the induction of ROS (exogenous mechanisms). Although generally considered as harmful by-products of oxygen metabolism, ROS also have important physiological functions in signal transduction and immunological response. In some cases, ROS can enhance the antitumor efficacy of chemotherapy [14,15]. The implications of ROS in cancer and cancer treatment are complex because ROS participate as signaling molecules in cell physiological processes of proliferation and survival. ROS can activate pro-tumorigenic signaling, enhance cell survival and proliferation, and result in DNA damage and genetic instability. However, ROS can also promote anti-tumorigenic signaling, initiating OS-induced tumor cell death [16].

The OS reflects the imbalance due to an excess of ROS or oxidants, or downregulation of antioxidant systems, or both. These conditions overcome the capability of cells to exert effective antioxidant responses [17]. Excessive ROS production may arise from mitochondria dysfunction or by the interaction between normal or excessive mitochondrial production with exogenous sources. The inner membrane of the mitochondria located near the ROS production site is prone to lipid peroxidation favoring a chain production process of more ROS. The first ROS directly produced from O_2_ (and the precursor of all other ROS) is the superoxide radical (O_2_•−). Dismutation of O_2_•− (spontaneous or mediated by superoxide dismutase (SOD)) generates hydrogen peroxide (H_2_O_2_), which in the presence of transition metals (Cu+ or Fe2+) can undergo Fenton reaction to generate the hydroxyl radical (HO•). Depending on the degree of the OS, it results in the modification of intracellular signal pathways or in functional damage mediated by macromolecular oxidation. Lipid peroxidation generates direct products, such as malondialdehyde (MDA), isoprostanes, and 4-hydroxynonenal. Protein oxidation can cause the fragmentation of amino acid residues, the formation of protein–protein cross-linkages or other protein adduct linkages, and oxidation of the protein backbone. Oxidative damage to DNA causes alterations in DNA bases. In addition, MDA can react with DNA to form DNA adducts [18].

OS secondary to neuronal mitochondrial DNA dysfunction can be induced in different ways by chemotherapy drugs. Mitochondria does not have DNA repair systems to repair mitochondrial DNA adducts produced by platinum compounds and the consequence is an impairment of mitochondrial and respiratory chain function [10]. Mitochondrial DNA is not directly damaged by taxanes, but its binding to mitochondrial β-tubulin may result in mitochondrial calcium release with induction of intracellular Ca dysregulation, in addition to swollen and vacuolated mitochondria linked to OS production [9,19]. Mitochondrial alteration by vinca alkaloids can be also induced by dysregulation of mitochondrial calcium signaling [10]. Furthermore, bortezomib modifies mitochondrial function, enhancing the expression of pyruvate dehydrogenase kinase 1 and lactate dehydrogenase A, resulting in decrease in oxidative phosphorylation (with decreased production of ATP molecules from the Krebs cycle in the mitochondria) and enhancement of aerobic glycolysis lactate [20].

The role of OS with carcinogenesis is well known, but not well understood. There is also a lack of understanding regarding the pathophysiology of OS generated during some anti-cancer treatments. Thus, additional studies are needed to understand these paradoxes in oncology, including those related to the controversial usage of antioxidants in the prevention or treatment of cancer [21]. From a time-based perspective, OS can be acute or chronic. Temporal moderate OS can boost cellular antioxidant capacities with positive, hormetic effects [22]. However, chronic OS may originate from deleterious effects in the main biomolecules, and induce DNA mutations and epigenetic changes that may impact the development and progression of many diseases including malignant alterations and cellular death [23]. Mechanistically, ROS can induce DNA double-strand breaks (DSBs) and are also able to oxidize nucleoside bases which can lead to G-T or G-A transversions.

The peripheral nervous system is exposed to the same chemotherapy action as tumor cells and chemotherapy can induce structural changes in peripheral nerves, such as neuronopathy, axonopathy, and/or myelinopathy, which contributes to the pathogenesis of CIPN [24]. However, it is not clear if the antitumor mechanisms of the action of chemotherapy are also implicated in CIPN, which involves nonproliferating neurons.

Several other pathologic mechanisms have been proposed, and probably different combinations of them occur for different drugs: (1) DNA damage and subsequent alterations, and the dysregulation of intracellular transcriptional and signaling pathways, particularly those associated with DNA repair; (2) drug cellular influx or efflux, particularly alteration of calcium homeostasis and ion channel functions; (3) alterations in metabolism, mitochondrial, and detoxication functions; (4) OS and associated-induced apoptosis, with several studies pointing at scavengers alterations associated with glutathione S-transferase; (5) local alterations of the immune system with local processes of neuroinflammation and microischemia with axonal degeneration [10,25,26].

Some of these proposed mechanisms are potentially supported by additional findings at the clinical level. Persistent CIPN by platinum-derivatives and taxanes has been associated with decreased levels of vitamin E and prealbumin, and increased levels of proinflammatory cytokines (IL-1, IL-6, IL-8, TNFα) [9,27]. An increased incidence of CIPN has also been associated with the addition of anti-angiogenic drugs [3].

TRP ankyrin-repeat 1 (TRPA1) is largely co-expressed with TRP vanilloid 1 (TRPV1), and they act as multimodal sensors of noxious stimuli [28]. TRPA1 is mainly expressed in a subset of C- or Aδ-fibers of sensory ganglia and nociceptors [28], and TRPA1-expressing afferents have been implicated in CIPN [29,30]. Several chemotherapy drugs may induce pain through a mechanism involving up-regulation of TRPA1 expression, such as carboplatin [31], oxaliplatin [29,32,33], paclitaxel [33], vinca alkaloids [34], thalidomide [35], or bortezomib [29]. As increased OS in CIPN can target the ion channel TRPA1 expressed by nociceptors [36], it has been proposed that antioxidants may be a novel therapeutic strategy to prevent CIPN [29]. There are experimental data that support the hypothesis that links the manipulation of the redox system and the reduction in pain mediated by TRPA1 up-regulation. For example, α-lipoic acid could completely prevent hypersensitivity if administered before the cytotoxic drug in a mouse model system [29], and the inhibition of NADPH oxidase subtype 4 decreased OS and decreased the upregulation of TRPA1 induced by oxaliplatin [37]. Ozone therapy could also potentially act by a similar mechanism [12,38].

Several chemotherapy drugs can lead to CIPN. Usually, the longest nerves are the first affected, maybe as a consequence of their higher metabolic requirements [24]. Symptoms are symmetric and start at the distal level, in the feet and hands, with further progression to the ankles and wrists following a “stocking and glove” distribution [24]. Symptoms are more often sensory than motor, although thalidomide and paclitaxel can show motor symptoms with higher probability than other chemotherapy agents. With the exception of vinca alkaloids, autonomic neuropathy is uncommon.

Below, we provide a short review of the pathologic mechanisms of CIPN production of the four most relevant drugs involved in CIPN: platinum, taxanes, vincristine, and bortezomib. Further details of these mechanisms are described in several recent reviews [2,9,10,26,39].

### 2.1. CIPN by Platinum Analogs

Platinum compounds include drugs such as cisplatin, oxaliplatin, and carboplatin. These are alkylating agents that are cell cycle-phase nonspecific, and can bind to proteins, RNA, and DNA, with inhibition of DNA synthesis and cell cycle, and induction of apoptosis. They are effective against different tumors (e.g., urological, gynecological, colon and rectum, lung, head and neck, and esophagus) in multidrug schemes or concurrent with radiotherapy. Some side effects of platinum compounds can be potentially dose-limiting, such as myelosuppression, nephrotoxicity, ototoxicity, and CIPN. The cumulative chronic CIPN is similar in all platinum compounds, with lower frequency and intensity in carboplatin. Usually, this CIPN is a symmetric, distal, sensory axonal peripheral neuropathy, with a “stocking and glove” distribution, and a slow and sometimes partial recovery. Additionally, oxaliplatin can also induce an acute, transient neuropathy in almost 90% of patients within a few hours of infusion, and from a few days to one week, and reappears with further infusions. This acute CIPN is a consequence of a transient impairment of ion channels and nerve hyperexcitability due to Na channel activation, and clinically is characterized by cold-induced distal or perioral paresthesias and pharyngolaryngeal dysesthesias. At a lower frequency (one-third of patients), the acute CIPN by oxaliplatin can also include motor symptoms, such as muscle cramps, jaw stiffness, difficulty swallowing, or visible fasciculations [39,40].

The bodies of sensory neurons located in the dorsal root ganglion seem to be the main target for platinum-induced peripheral neuropathy, where the apoptosis level correlates with the platinum-DNA binding. The formation of the DNA adducts is two to three times lower by oxaliplatin than by cisplatin and can be inhibited in vitro by caspase inhibitors. Damage to mitochondrial DNA leads to its functional impairment and further oxidative stress mediated by redox-sensitive TRPA1 channels [10]. Additional damage in other structures within the peripheral nervous system can be also involved in CIPN by platinum compounds, such as satellite cells, Schwann cells, and neuronal and glial cells in the spinal cord or axons [26,39].

### 2.2. CIPN by Taxanes

Taxanes (paclitaxel, docetaxel, cabacitaxel, and nab-paclitaxel) are antimicrotubule agents that are used to treat a variety of tumors. Both are associated with a predominantly sensory neuropathy, although paclitaxel appears to be more neurotoxic than docetaxel (overall incidence of any grade neuropathy is 60% versus 15%) [41]. Cabazitaxel is a semisynthetic taxane that is approved for the treatment of advanced prostate cancer. It appears to be less neurotoxic than either paclitaxel or docetaxel. Peripheral neuropathy of any grade is reported in 13% to 17% of treated patients, but less than 1% are severe [42]. Taxanes typically cause a sensory neuropathy, mostly affecting small diameter sensory fibers, described as paresthesia, numbness, neuropathic pain or altered proprioception, and loss of ability, predominantly in feet and/or hands (stocking-and-glove distribution). Alteration tends to be distal symptoms (in fingers) initially and further spread centrally, in a “dying back” way. Deficits in motor function are less common, but can be present in severe cases [2,19].

The mechanism of taxane-induced peripheral neuropathy appears to be related to excessive tubulin polymerization and further disruption of microtubules of the mitotic spindle, which interferes with axonal transport, macrophage activation in both the dorsal root ganglia and peripheral nerve, and microglial activation within the spinal cord [43]. In addition, taxanes evoke a “dying back” process, starting from distal nerve endings followed by effects on Schwann cells and other neuronal cells, which is an essential microtubule-based process that moves cellular components over long distances between neuronal cell bodies and nerve terminals [43].

The most important triggering factor for taxane-induced peripheral neuropathy is the cumulative dose; the neurotoxic threshold for paclitaxel is 1 g/m^2^, whereas it is 0.4 g/m^2^ for docetaxel. Most patients can expect symptom improvement or resolution within 3–6 months after the discontinuation of treatment, but more severe cases tend to be less likely to resolve [19,25].

The caspases are a family of cysteine proteases that signal apoptosis. Concerning CIPN, the activation of caspases contributes further to neuron damage. Paclitaxel produces a range of damaging effects via caspase signaling, including mitochondrial damage, ROS production, and neuron-level apoptosis. The paclitaxel induction of ROS mitochondrial production can worsen mitochondrial function [2,9,44].

### 2.3. CIPN by Vincristine

Vincristine is the most neurotoxic of the vinca alkaloids. These drugs are useful agents for many cancers, such as leukemias, lymphomas, and small cell lung cancer. CIPN by Vincristine is an axonal neuropathy resulting from disruption of the microtubules within axons and interference with axonal transport [2]. Vincristine cause changes to large axons and dorsal root ganglion neurons, which leads to Wallerian degeneration, the altered activity of ion channels, and the hyperexcitability of peripheral neurons. In addition, the inhibition of polymerization in microtubules affects axonal transport, which leads to distal axonopathy. In consequence, there is an alteration in the excitability of peripheral neurons, whereas the attraction and activation of immune cells by vincristine causes the release and elevation of pro-inflammatory cytokines, resulting in neuroinflammation [2].

The neuropathy involves both sensory and motor fibers, although small sensory fibers are especially affected. Virtually all patients receiving vincristine have some degree of neuropathy that can be dose-limiting toxicity. Although vinca alkaloids do not readily cross the blood–brain barrier, they can act on the cell body of peripheral nerves. Symptoms usually appear within the first 3 months of treatment. The earliest symptoms are usually symmetric paresthesias in the fingertips and feet, with or without pain, muscle cramps, and/or mild distal weakness. Other symptoms include muscle weakness, including wrist extensors, dorsiflexor weakness, and cramping. Vincristine may also produce mononeuropathies, involving cranial nerves and the autonomic system. CIPN by vincristine often develops after several weeks of treatment, although it can occur after the first dose. These symptoms are usually observed after cumulative doses higher than 4 mg/m^2^, although sensory symptoms and painful paresthesias tend to occur earlier, at lower doses, and distal weakness typically occurs at higher doses, but may occur nonetheless [2,9].

### 2.4. CIPN by Bortezomib

Bortezomib, carfilzomib, and ixazomib are proteasome inhibitors that are used in the treatment of lymphoid tumors and multiple myeloma and have been associated with peripheral neuropathy. Peripheral neuropathy appears to be less common and less severe with ixazomib and carfilzomib compared with bortezomib.

The action mechanism of bortezomib is the reversible inhibition of the chymotrypsin-like activity of the 20S proteasome core in the 26S proteasome complex, which degrades ubiquitinated proteins that regulate the intracellular concentration of unneeded or damaged proteins. In this way, misfolded and unrequired proteins to be degraded by the proteasome accumulate in the endoplasmic reticulum (ER), which promotes the overproduction of ROS leading to ER stress-related apoptosis and cell death due to DNA damage. The inhibition of proteosome by bortezomib was initially associated with the inhibition of the NF-κB pathway. However, the NF-κB activity (at least in multiple myeloma cells) is mediated via two distinct pathways, canonical and non-canonical, which show opposing activity after bortezomib treatment, and full knowledge of the NF-κB regulation is not complete yet [45].

Finally, other studies have associated CIPN by bortezomib with interference with transcription, nuclear processing, and cytoplasmic translation of mRNAs in dorsal root ganglia neurons, with further damage of large and C-fibers [25,46]. Damage to dorsal root ganglia has also been associated with infiltration by inducible nitric oxide synthase (iNOS)-expressing inflammatory macrophages [47].

CIPN is one of the most significant nonhematologic toxicities of bortezomib. CIPN by bortezomib is usually a painful, sensory axonal neuropathy, in a stocking-glove distribution with burning dysesthesias. When this neuropathy occurs, it can interfere with the quality of life and the performance of activities of daily living, and it may force dose modification and/or treatment discontinuation. The incidence and severity of peripheral neuropathy depend on the cumulative drug dose, the dosing schedule, and the route of administration. With twice-weekly schemes of bortezomib treatment, CIPN has been described in more than 60% of patients, with 15% of severe (grade 3 or grade 4) neuropathy. A lower incidence of CIPN has been described with once-weekly schemes of treatment (<5% grade 3 or grade 4 neuropathy) and with subcutaneous rather than intravenous administration. Bortezomib can also induce distal motor neuropathy in lower members (which could be immune-mediated), in addition to reversible posterior encephalopathy syndrome (which could be related to dysregulation of cerebral vasomotor autoregulation) [48].

## 3. Summary of Prophylaxis and Treatment of CIPN

There are no agents with highly relevant effects for the prevention or treatment of CIPN [8].

No agents are recommended for prevention of CIPN. Several substances have been evaluated, many with an antioxidant effect (e.g., acetylcysteine, amifostine, glutathione, Org 2766, or vitamin E), but without conclusive results. Here, we would like to mention the ongoing studies of calmangafodipir. This is a mitochondrial manganese superoxide dismutase mimetic (acts as an antioxidant) which is currently under randomized clinical trials to evaluate its potential for preventing the development of oxaliplatin-induced peripheral neuropathy [49].

The recent ASCO guideline on the prevention and management of CIPN [8] does not recommend for the prevention of CIPN oxcarbazepine or venlafaxine (despite the promising initial reports) among several other agents, such as carbamazepine, gabapentin, pregabalin, and the antioxidants previously mentioned (type of recommendation: evidence-based, no benefits; evidence quality: intermediate; strength of recommendation: moderate). Furthermore, there is a recommendation against the use of acetyl-L-carnitine for the prevention of CIPN in patients with cancer (type of recommendation: evidence-based, harms outweigh benefits; evidence quality: high; strength of recommendation: strong) [8].

For the treatment of CIPN in patients who have completed neurotoxic chemotherapy, duloxetine is the only agent that has provided appropriate evidence to support its use. Duloxetine could be offered to patients with painful CIPN, with low harm but low benefit (type of recommendation: evidence-based, benefits equal harm; evidence quality: intermediate; strength of recommendation: moderate) [4,7,8]. Duloxetine is an antidepressant drug that, in some countries (e.g., Spain), also has an indication for secondary pain in diabetic-induced peripheral neuropathy, but not for CIPN.

Outside the context of clinical trials, there is no recommendation for the use of other drugs/interventions for the treatment of stablished CIPN, including gabapentin, pregabalin, tricyclic antidepressants, oral cannabinoids, or topical gel treatment containing baclofen, amitriptyline HCL, plus/minus ketamine [8]. Preliminary evidence suggests a potential beneficial effect in the management of CIPN by exercise, acupuncture, and scrambler therapy. However, further studies with larger sample sizes are required to confirm efficacy and clarify the potential risks [8].

Opioids have been frequently used for the management of pain secondary to CIPN, although its beneficial effect has not been demonstrated in this condition. In addition, we should keep in mind that in cancer-free patients it is recommended to avoid opioid prescriptions [50].

Due to the limited therapeutic options, in patients with symptomatic CIPN, some authors have considered that it could be reasonable to offer treatments with useful effects in neuropathic pain [7], although the potential beneficial and adverse effects should be widely discussed with patients. Regardless, it is clear that further research about the prevention and treatment of established CIPN is required [1].

## 4. Modulation of Oxidative Stress by Ozone Therapy

Ozone (O_3_) is a molecule formed by three atoms of oxygen. It is more reactive, less stable, and more soluble (10 times) in water and plasma than oxygen (O_2_). Ozone is a strong oxidant, ranking third after fluorine and persulfate. The medical use of ozone (ozone therapy) represents the use of a gas mixture of O_3_/O_2_, from a medically certified device with medical-grade oxygen, by a medically trained doctor using a specific medical protocol in the treatment of different pathologies. Ozone therapy is considered a medical treatment. For every application there are defined dose ranges and different administration routes depending on the pathology to be treated. In general terms, O_3_ is generated in situ because of the short half-life (at 20 °C the O_3_ concentration is halved within 40 min, at 30 °C within 25 min). Regular clinical O_3_ concentrations range from 10 to 60 µg/mL of a mixture of O_3_ (0.5–0.05%) and O_2_ (95–99.5%) [51,52,53].

The benefit of ozone in medicine depends on the dose, the method of the administration, and the concentration of exposure. For example, the inhalation of ozone causes serious damage and chronic oxidative stress, induces the gene transcription of pro-inflammatory cytokines, its receptor, inflammatory proteins, negative regulation of type 1 Interferon, and response to viral infections pathways, and is associated with increase in blood pressure [54,55]. However, the administration of ozone to a target with better antioxidant capacity (e.g., blood, rectum, and colon mucosa), exposed to a small and precise dose of ozone, can modulate the endogenous antioxidant system and aids in the control of different pathological conditions [56].

We have previously described how the modulation of OS by ozone can lead to an enhancement of antioxidant mechanisms from a wide perspective, in addition to focusing on chemotherapy-induced side effects [12]. Autohemotherapy and rectal insufflation are the principal methods of clinical administration when looking for a systemic effect. However, this is an indirect effect. By these routes, ozone does not enter the blood circulation and is not able to reach any specific target tissues. A relevant percentage of ozone will be removed by the antioxidants of the medium. Nonetheless, the remaining ozone will interact with biomolecules (e.g., unsaturated fatty acids from cell membranes in intestinal mucous, rectal administration or blood cells, and auto-hemotherapy), generating secondary byproducts (e.g., aldehyde as 4-hydroxynonenal (4-HNE) and H_2_O_2_). These substances act as second messengers and induce a further adaptive antioxidant in a hormetic dose–response relationship [51,53,57]. Thus, the action mechanism of systemic ozone therapy is a result of an “indirect” effect. Ozone dose/response does not follow a linear relationship, but a hormetic relationship: very low concentrations can have no effect and very high concentrations can lead to effects contrary to those produced by lower/middle concentrations [58]. Hence, there are established therapeutic windows for all methods of administration, based on the clinical experience of the main school of ozone therapy worldwide and on the main current scientific literature [52].

Medical ozone has a pro-drug effect, and most relevant mediators (4-HNE and H_2_O_2_) induced by ozone play an important role in the action mechanisms involved during medical applications [59,60].

**Figure 1:**
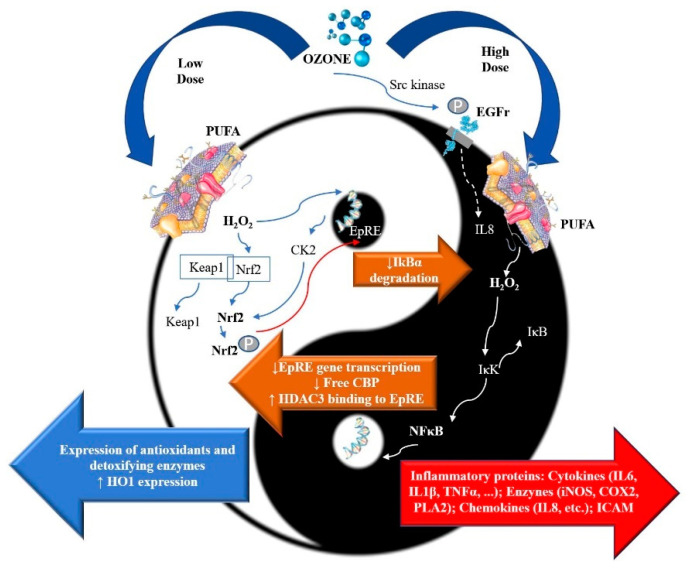
Hypothetic effects of ozone mediators on Nrf2 and NfκB pathways. After administration, ozone reacts with biomolecules, including polyunsaturated fatty acids (PUFA) or plasma membrane, producing hydroperoxides, aldehydes, and H_2_O_2_ [60]. H_2_O_2_ can enter the cytoplasm of mononuclear cells and modulate nuclear factor NF-κB/Nrf2 pathways. Casein kinase 2 (CK2), CREB binding protein (CBP), cyclooxygenase-2 (COX-2), Electrophile-responsive elements (EpRE), Epidermal growth factor receptor (EGFr), Heme-oxygenase-1 (HO-1), Histone deacetylase 3 (HDAC3), Inducible nitric oxide synthase (iNOS), Interleukin-1β (IL-1 β), Interleukin-6 (IL-6), Interleukin-8 (IL-8), Intracellular adhesion molecule (ICAM), IκB kinase (IKK), Kelch-like ECH-associated protein 1 (Keap1), Nuclear erythroid 2 related factor 2 (Nrf2), Phospholipase A2 (PLA2), Tumor necrosis factor-α (TNF-α).

After its administration, systemic ozone reacts with biomolecules, including polyunsaturated fatty acids (PUFA) or plasma membrane, producing hydroperoxides, aldehydes, and H_2_O_2_ [60]. At low doses of ozone (between therapeutic ranges), hydrogen peroxide inside the cell acts as a second messenger and modulates nuclear factor NF-κB/Nrf2 pathways. This fact is already demonstrated in cells [56], ex vivo experiments [60], and clinical trials [61]. The fact that H_2_O_2_ generated by ozone treatment can act as an activator of the Nrf2 pathway has been recently described [56,62,63]. H_2_O_2_ increases the expression of casein kinase 2 (CK2), possibly acting at the nucleal level [61]. H_2_O_2_ also dissociates the complex between Nrf2 and Kelch-like ECH-associated protein 1 (Keap1), releasing Nrf2. The free Nrf2 is phosphorylated by CK2, leading to its nuclear translocation and transcriptional activation. Activated Nrf2 bind to electrophile-responsive elements (EpRE), promoting the expression of different detoxifying, antioxidant, and cytoprotective enzymes, such as SOD, catalase, heme-oxygenase-1 (HO-1), and NADPH quinone oxidoreductase (NQO1), and increased synthesis of glutathione, NADPH, and multidrug transporters. In contrast, high doses of ozone induce epidermal growth factor receptor (EGFR) phosphorylation, through cytosolic tyrosine kinase Src, which further modulates O_3_-induced IL-8 expression [64]. Moreover, ozone-induced H_2_O_2_ induces activation of IκB kinase (IKK), which causes phosphorylation of inhibitors of NF-κB and IκB, and hence targets the latter for polyubiquitination-mediated proteasomal degradation, and results in the release of NF-κB. NF-κB migrates into the nucleus, binds with the κ region of the genome, and causes the transcription of proinflammatory mediators, such as IL-1, IL-6, TNF-α, cyclooxygenase-2 (COX-2), Phospholipase A2 (PLA2), intracellular adhesion molecule (ICAM), and inducible nitric oxide synthase (iNOS). Furthermore, these two pathways are proposed to inhibit each other at their transcription level via protein–protein interactions or through secondary messenger effects. The Nrf2 pathway inhibits the activation of the NF-κB pathway by increasing antioxidant defenses and HO-1 expression, which efficiently neutralizes ROS and detoxifies toxic chemicals, and hence reduces ROS-mediated NF-κB activation. The Nrf2 pathway also inhibits NF-κB-mediated transcription by preventing the degradation of IκB-α. Similarly, NF-κB-mediated transcription reduces Nrf2 activation by reducing the EpRE gene transcription and decreases free CREB binding protein (CBP) by competing with Nrf2 for CH1-KIX domain of CBP. NF-κB also enhances the recruitment of histone deacetylase 3 (HDAC3) to the EpRE region by binding to Mafk, and hence interferes with the transcriptional facilitation of Nrf2 [65] (Figure 1).

One of the main mediators of the ozone response, H_2_O_2_, emerges not as an inducer of NF-kB, but as modulator of the activation of the NF-κB pathway by other agents. Therefore, H_2_O_2_ is a fine-tuning regulator of NF-κB-dependent processes, as exemplified by its dual regulation of inflammation [66]. It has been hypothesized that a therapeutic dose of O_3_ inhibits the NF-κB signal, reducing inflammation [67]. Moreover, a high dose of O_3_ promotes inflammation by the activation of the NF-κB pathway [64]. Additionally, the inhibition of the NF-κB pathway could potentially downregulate iNOS [68] because, for bortezomib treatment, it has been described that most of the infiltrating macrophages in the dorsal root ganglia are iNOS-expressing inflammatory macrophages [47].

Probably the key effect of ozone therapy is to restart the balance of NF-κB/Nrf2. This mechanism has been shown in an in vitro model of doxorubicin-induced damage in human skin fibroblast cells and human fetal cardiomyocytes [69]. In this study, it was observed that the restoration of the equilibrium NF-κB/Nrf2 was essentially reached by the preservation of the level of Nrf2 in the ozone-treated group [38]. Similar effects were observed in an experimental model of tubulointerstitial injury in rats with adenine-induced chronic kidney disease. In this case, ozone therapy attenuates the damage via a mechanism that mediates the modulation of Nrf2 and NF-κB [70]. In addition, the re-analysis of clinical studies of multiple sclerosis treated rectally with ozone for one month [61], confirm the modulating effect of O_3_ in the balance of Nrf2/NF-κB [38].

Another interesting potential mechanism could be the modulation of transforming growth factor β1 (TGF-β1). TGF-β1 decreases the sensorial hypersensitivity associated with nervous damage [71]. Experimental studies inducing damage in the sciatic nerve described an increase in miRNA (specifically miR-30c-5p) in cerebrospinal fluid and plasma. This miRNA can decrease the expression of TGF-β1 and be associated with neuropathic symptoms [72]. Indeed, the inhibition of miR-30c-5p increased TGF-β1 and decreased neuropathic pain and suggests the therapeutic potential of this approach [72]. Thus, the modulation of TGF-β1 by ozone therapy could be another of the action mechanisms of ozone. Airway exposition to ozone is dangerous, and it has been described that ozone-induced airway fibrosis is mediated by an increase in TGF-β1 expression [73]. However, appropriated ozone treatment by systemic routes (avoiding airway inhalation) can increase TGF-β1 levels in blood [74], which could induce potential beneficial effects on neuropathic pain [71,75].

Additional mechanisms of ozone in the control of pain may involve the inhibition of purinergic receptors P2 × 3 and P2 × 7 [76]. The reduction in autophagy and autophagy promoters LC3B and Beclin 1 in nerves blocks the apoptosis, and inactivates caspase 3 and the signals of phosphodiesterase 2A (PDE2A) and nuclear factor kB p65 (NF-kB p65) [77]. In addition, local applications of ozone in the peri-sciatic nerve activates 5′-adenosine monophosphate (AMP)-activated protein kinase (AMPK) to attenuate chronic constriction and injury-induced neuropathic pain [78].

## 5. Ozone Therapy in Chemotherapy-Induced Neurologic Symptoms and CIPN

Ozone therapy can induce an adaptive modulation against OS, inflammation, and ischemia/hypoxia. This bodes well for a potential beneficial effect of ozone in CIPN when these mechanisms are involved. Recently, we summarized the main experimental research describing the potential of ozone treatment to prevent or treat several chemotherapy-induced side effects [12]. However, research focused on chemotherapy-induced neurologic complications or CIPN are even less common. Here, we describe a few relevant related studies that offer potential support for further research about ozone in CIPN.

An experimental study evaluated rats intraperitoneally injected with 5 mg/kg/day cisplatin for 3 days to produce cisplatin-induced ototoxicity, which was confirmed by test with distortion product otoacoustic emissions. Rats were randomized to (1) no treatment (control group); (2) ozone by “rectal” insufflation; (3) ozone by “rectal + intratympanic” insufflation. Rectal and intratympanic insufflations consisted of 2.3–3 mL of O_3_/O_2_ gas at a concentration of 60 µg/mL administered once per day for 7 days. Ototoxicity was significantly lower (*p* < 0.05) in ozone groups than the control group, with (1) partial recovery of audition and lower distortion of product otoacoustic emissions, and (2) lower histopathological damage in the outer hair cell of the inner ears. However, the addition of intratympanic insufflation did not provide further benefit when compared with rectal insufflation of ozone alone [79].

A study on rats assessed the effect of ozone in drug-induced diabetic neuropathy. Four weeks after the induction of diabetes by a single intraperitoneal injection of streptozotocin, the right sciatic nerves were removed. Compared with the “diabetic group without ozone and without insulin”, the diabetic groups treated with ozone, or with insulin, or with ozone + insulin, showed higher amplitudes of conduction velocity and compound action potential, higher total antioxidant status, lower total oxidative status, and lower OS index. Thus, this study showed that ozone treatment partially prevented drug-induced neuropathy, and this effect was mediated by modulation of OS [80].

It has been also described that ozone can offer neuroprotective effects after an injury of cutting of the sciatic nerve. In a large experimental study with one hundred rats, a transverse cut injury to the sciatic nerve was produced, with further reparation of nerve stumps. Compared with the group without ozone treatment, the group with two months of intraperitoneal ozone treatment (5 mL at a concentration of 35–40 μg/mL) showed more myelinated nerve fibers under electron microscopy, and an increase in plasma antioxidants (SOD, CAT, GSH-Px). Although the pathogenic mechanism in this study was not drug-induced, and could not be directly applicable for CIPN, we believe it is of interest to note the described enhancement of damaged-nerve regeneration by ozone treatment [81].

Finally, we describe our experience in a small group of patients with chronic neuropathic pelvic pain secondary to cancer treatments (chemotherapy, radiotherapy, and surgery) [82,83]. In this group, cancer treatment included radiotherapy in five patients, chemotherapy in four, and surgery in two. There was complete tumor response after cancer treatment, but patients experienced refractory chronic pelvic pain after several months of conventional treatments. Pain level, according to the Visual Analog Scale (VAS) was 7.8 ± 2.1 before the commencement of ozone therapy by local and rectal insufflation. There was a significative (*p* < 0.05) and clinically relevant decrease (>5 points in VAS) pain reduction after the first three months of ozone treatment [82], at the end of treatment, and nine months after the end of ozone therapy. Five of six patients were able to decrease or even discontinue analgesic intake requirements [83].

## 6. Ozone Therapy on Oxaliplatin-Induced Peripheral Neuropathy: A Focused Clinical Trial

Oxaliplatin is a platinum-based compound used in the treatment of several gastrointestinal tumors, including colorectal cancer, one of the most common tumors in both men and women. As previously stated, CIPN by oxaliplatin may be dose-limiting and sometime can lead to interruption of the programmed scheme of chemotherapy. Currently, a double blinded randomized controlled trial (RCT) is ongoing at our hospital. This trial was approved by the regional Ethics Committee and the Spanish Medicine Agency, and it was registered with EudraCT number 2019-000821-37 and ClinicalTrials.gov Identifier NCT04299893. Here, we summarize the most relevant characteristics about this focused clinical trial, although further details are available at https://clinicaltrials.gov/ct2/show/NCT04299893 (accessed on 30 January 2021).

The main objective of this clinical trial is to evaluate the effectiveness and cost-effectiveness of adding ozone therapy to the clinical management of patients with colorectal cancer and pain secondary to oxaliplatin-induced peripheral neuropathy. The primary outcome measures are: (1) change from baseline in “average pain” according to the Brief Pain Inventory-Short Form (BPI-SF) at the end of the 16 weeks of ozone therapy by rectal insufflation, and (2) direct hospital costs.

Changes from baseline will also be assessed via: (1) other pain scales, such as the Neuropathic Pain Symptom Inventory (NPSI); (2) several quality-of-life questionnaires, such as QLQ-C30 (from the European Organization for Research and Treatment of Cancer), SF-36, and the EQ-5D-5L; (3) anxiety and depression questionnaires; (4) assessment by hyperspectral imaging of painful areas; (5) several biochemical parameters, with special focus on proinflammatory cytokines (TNFα and several interleukins) and OS parameters such as SOD, GSH-Px, GSH, CAT, MDA, peroxidation potential, and redox index. The study is open to potential collaborations, and further parameters could be evaluated.

The main inclusion criteria are (1) adults at least 18 years old; (2) cancer of colon and rectum at any stage, with treatment including oxaliplatin, chemotherapy finished at least 3 months before, and life expectancy higher than 6 months; (3) clinical diagnosis of “painful” CIPN, with toxicity Grade 2 or higher according to the Common Toxicity Criteria for Adverse Events (CTCAE) from the National Cancer Institute of EEUU, v.5.0, for at least 3 months, and without inclusion of new treatments for pain and/or neuropathy during the last month; (4) “average pain” of 4 or higher according to the Brief Pain Inventory-Short Form (BPI-SF) (range from 0 to 10) for at least 3 months following chemotherapy completion.

As a mismatch between doctors and patients in the assessment/gradation of the neuropathy has been described [84], more relevance is currently being given to symptom evaluation scales focused on the patients’ own assessment [84,85]. Our research will be focused on the effect of ozone therapy on the analytical and symptomatic evolution of patients with CIPN. Thus, the course of this RCT will allow us to explore whether there is a relationship between the basal levels (or evolutive changes) in oxidative stress parameters with new approaches to assess tissue alteration, such as hyperspectral imaging (HSI) or the symptoms and quality of life self-reported by the patients.

## 7. Discussion

CIPN is an important long-term issue due to the improvement in the overall survival of cancer patients, mainly for those with breast, colon, and rectum carcinomas. CIPN can lead (temporarily or permanently) to dose reduction or interruption of cancer treatment, with an adverse impact on cancer outcome. Expanding the understanding and use of pain management options for cancer patients, and identifying strategies to mitigate side-effects of cancer treatment, are urgent areas of research in oncology [1].

Although the mechanisms that lead to CIPN are not well understood, several alterations have been described, and many of these are related to increased local ROS production and OS.

Given the absence of effective preventive agents, clinicians should carefully assess the benefits of agents known to cause CIPN against the risks of developing long-term irreversible neuropathy, especially among patients with underlying neuropathy and with conditions that predispose to neuropathy (e.g., diabetes mellitus and/or a personal or family history of hereditary neuropathies). Clinical studies have described that miR-30c-5p levels are related with neuropathic pain intensity [72]. This study opens the possibility in the future of using micro-RNA as a clinical marker of this syndrome.

The absence of effective treatment for established CIPN, except the limited effect of duloxetine, encourages the exploration of new drugs and approaches in RCT. Treatment with appropriated concentrations of ozone can induce an adaptive response of tissues, with further enhancement of antioxidant systems. One of the keys is the induction of Nrf2, which can upregulate antioxidant response elements and downregulate NF-κB with a potential subsequent decrease in proinflammatory cytokines, iNOS, and other substances involved in CIPN. Furthermore, a deleterious effect of ozone treatment on the oncological process would not be expected, as supported by the few clinical studies about ozone therapy on the side effects of cancer treatments with long follow-up [83,86,87]. We have previously provided detailed justification about this potential issue [12,88].

An RCT is ongoing to evaluate the effect of ozone therapy in patients treated with oxaliplatin and with established pain secondary to CIPN. The trial will also evaluate the potential relationship of OS parameters with the effect of ozone treatment and with several symptoms associated with CIPN. The different aims of our RCT include the evaluation of the potential relationship of OS with three innovative topics. One of these is the potential beneficial effect of ozone therapy based on its modulation of OS, which has been detailed in this review. The other two potential relationships to evaluate are those with HSI and with the anxiety and depression of these patients.

HSI is a new emerging imaging modality based on a combination of spectroscopy and digital imaging that has been widely employed in the field of medical research to analyze in vivo and ex vivo cancer tissue [89] or histological samples [90], among others. HSI is a non-contact and label-free imaging modality able to obtain significantly more information across the electromagnetic spectrum about the captured scene, within and beyond the visual spectral range, than conventional optical imaging modalities [89]. Using this increased amount of information, it is possible to identify and differentiate among the materials and substances presented in the scene, providing diagnostic information regarding the tissue morphology, physiology, and composition. Several studies support its potential use in the assessment of hypoxia and ischemia [91,92,93]. In addition, spectroscopic methods have been employed to perform a label-free detection of hydrogen peroxide-induced OS and the protective effect in human retinal pigment epithelium cells [94], and to assess metabolic status and oxidative stress in human-induced pluripotent stem cell-derived cardiomyocytes [95]. HSI has been previously employed as a method for monitoring therapies based on cold atmospheric plasma, a controllable source for reactive species, such as H_2_O_2_ and NO_2_- (in addition to neutral particles, electromagnetic field, and UV radiation) [96]. HSI has been able to demonstrate, in vivo, the associated effects on microcirculation in the treatment of head and neck cancer and wound healing [97]. In this sense, we consider it of special interest to explore the potential relationship of HSI with OS parameters and its capabilities to measure cellular and tissue properties, in addition to their functional behavior, to evaluate and assess the efficacy of ozone therapy in reducing the pain induced by CIPN.

Depressive and anxiety disorders share biological and environmental aspects with chronic pain disorders. Over the past decade, OS has emerged as a major cause of these psychiatric disorders [98,99]. It is known that the brain is more vulnerable to OS than any other organ in the body because of its higher oxygen consumption, higher lipid content, and weaker antioxidative defense [100]. As a result of increased OS, the activation of proinflammatory signaling pathways also contributes to the pathogenesis of depression. This is due to the fact that the imbalance between ROS and antioxidative defenses leads to deregulation of brain functions and abnormalities in neuronal signaling [101,102]. It is well established that patients with depression and anxiety have higher plasma levels of C-reactive protein and proinflammatory cytokines (e.g., TNFa, IL-1a, IL-1b, IL-4, IL-5, IL-6, IL-12, and interferon). Some studies have revealed that increased levels of inflammatory markers combined with OS are a ubiquitous characteristic of depressive disorders [98,103], and these factors can decrease neurogenesis and increase neurodegeneration [99]. Our ongoing RCT will evaluate the relationship of anxiety and depression with pain and OS in patients with CIPN, and with the changes in basal levels after ozone treatment.

## 8. Conclusions

CIPN is a common side effect of cancer treatment with a potential impact on the success of the oncologic therapy and a high impact on the quality of life of patients. There is no evidence of clinically relevant prophylactic or therapeutic approaches. Further research is required to better understand the role of OS in CIPN and the clinical role of its modulation. Appropriate use of ozone is an effective adjuvant therapy that can modulate OS, although randomized clinical trials are urgently required to establish its potential benefit in CIPN. A related trial is ongoing.

## Data Availability

Data sharing is not applicable to this article.

## Abbreviations

4-HNE	4-hydroxynonenal
AMPK	5′-adenosine monophosphate (AMP)-activated protein kinase
ASCO	American Society of Clinical Oncology
BPI-SF	Brief Pain Inventory-Short Form
CAT	catalase
CBP	CREB binding protein
CIPN	chemotherapy induced peripheral neuropathy
CK2	casein kinase 2
CIT	chemotherapy-induced toxicity
COX-2	cyclooxygenase-2
CTCAE	Common Toxicity Criteria for Adverse Events
EGFR	epidermal growth factor receptor
EpRE	electrophile-responsive elements
ER	endoplasmic reticulum
GSH-Px	glutathione peroxidase
H_2_O_2_	hydrogen peroxide
HDAC3	histone deacetylase 3
HO•	hydroxyl radical
HO-1	heme oxygenase-1
HSI	hyperspectral imaging
ICAM	intracellular adhesion molecule
IKK	IκB kinase
IL	interleukin
iNOS	inducible nitric oxide synthase
Keap1	Kelch-like ECH-associated protein 1
MDA	Malondialdehyde
miRNA	microRNA
NF-κB	nuclear factor kappa B
NPSI	Neuropathic Pain Symptom Inventory
NQO1	NADPH quinone oxidoreductase
Nrf2	nuclear factor erythroid 2-related factor
O_2_	oxygen
O_2_•−	superoxide anion radical
O_3_	ozone
OH•	hydroxyl radical
OS	oxidative stress
PDE2A	phosphodiesterase 2A
PLA2	phospholipase A2
PUFA	polyunsaturated fatty acids
RCT	randomized controlled trial
ROS	reactive oxygen species
SOD	superoxide dismutase
TGF-β1	transforming growth factor β1
TNF-α	tumor necrosis factor-alpha
TRP	transient receptor potential channel
TRPA1	transient receptor potential ankyrin-repeat 1 channel
VAS	Visual Analog Scale
